# Effects of triethylene glycol dimethacrylate (TEGDMA) on the odontoclastic differentiation ability of human dental pulp cells

**DOI:** 10.1590/1678-7757-2016-0626

**Published:** 2017

**Authors:** Zeynep Öncel Torun, Deniz Torun, Barış Baykal, Ali Öztuna, Fatih Yeşildal, Ferit Avcu

**Affiliations:** 1 Balgat Oral and Dental Health Center, Ankara, Turkey.; 2 University of Health Sciences, Gulhane Faculty of Medicine, Department of Medical Genetics, Ankara, Turkey.; 3 University of Health Sciences, Gulhane Faculty of Medicine, Department of Histology and Embryology, Ankara, Turkey.; 4 Diyarbakır Selahaddin Eyyubi Public Hospital, Department of Medical Biochemistry, Diyarbakır, Turkey.; 5 Memorial Ankara Hospital, Ankara, Turkey.

**Keywords:** Human dental pulp cell, Odontoclast, OPG, RANKL, TEGDMA

## Abstract

**Objectives:**

The primary purpose of this study was to examine the effects of triethylene glycol dimethacrylate (TEGDMA) on odontoclastic differentiation in the dental pulp tissue.

**Material and Methods:**

The effects of different TEGDMA dosages on the odontoclastic differentiation capability of dental pulp cells were analyzed *in vitro* using the following methodologies: i) flow cytometry and tartrate-resistant acid phosphatase (TRAP) staining; ii) apoptotic effects using Annexin V staining; iii) mRNA expression of osteoprotegerin (OPG) and receptor activator of nuclear factor (NF)-kB ligand (RANKL) genes by quantitative Real-time PCR (qRT-PCR); and iv) OPG and RANKL protein expression by enzyme-linked immunosorbent assay (ELISA).

**Results:**

TEGDMA caused relatively less odontoclastic differentiation in comparison with the control group; however, odontoclastic differentiation augmented with increasing doses of TEGDMA (p<0.05). The mRNA and protein expression of OPG was lower in TEGDMA treated pulp cells than in the control group (p<0.05). While the mRNA expression of RANKL remained unchanged compared to the control group (p>0.05), its protein expression was higher than the control group (p<0.05). In addition, TEGDMA increased the apoptosis of dental pulp cells dose dependently.

**Conclusions:**

TEGDMA reduced the odontoclastic differentiation ability of human dental pulp cells. However, odontoclastic differentiation ratios increased proportionally with the increasing dose of TEGDMA.

## Introduction

Triethylene glycol dimethacrylate (TEGDMA) is a resin monomer widely used in the composition of dentin bonding agents and composite resins to restore teeth structures impaired by caries and/or fractures. However, resin monomers can be released into the oral environment and can trigger hazardous biological effects on oral tissues^2^. The release of the resin monomers due to degradation and incomplete polymerization can occur hours or days after the treatment^7^. Due to its hydrophilic nature, hydrolysis plays an important role in the degradation processes of TEGDMA^24^. Chemical interactions with oral fluids and mechanical influences may also cause the degradation of resin monomers^26^. Direct contact or diffusion of resin monomers through the dentinal tubules creates ways of interaction between dental pulp tissue and resin monomers. Dentin thickness and the severity of caries lesions are important factors in determining the amount of resin monomers interacting with dental pulp tissue^6^ ^,^ ^12^. TEGDMA has been reported to cause cytotoxicity, impaired cellular functions, pulpal inflammatory responses, and changes in the immune system^2^ ^,^ ^6^ ^,^ ^14^ ^,^ ^19^ ^,^ ^27^. In addition, TEGDMA may reduce the mineralization capacity of dental pulp cells by decreasing the expression of the mineralization related genes^11^. These findings suggest that TEGDMA interacts with living cells by influencing different biological pathways and causing adverse effects.

Odontoclasts are key regulators controlling the eruption of deciduous teeth, and they show similar biological features to osteoclasts^22^ ^,^ ^25^. Besides, odontoclasts have important functions in pathological resorption of permanent teeth^32^. OPG/RANK/RANKL signaling pathway is known to play a key role in the differentiation of osteoclasts, which is strictly controlled by osteoblasts^29^. Osteoblasts/stromal cells express the receptor activator of nuclear factor (NF)-kB ligand (RANKL) in response to cytokines like 1ɑ,25-dihydroxyvitamin D_3_ (lɑ,25(OH)_2_D_3_), parathyroid hormone (PTH), IL-6, and IL-11. The activation of RANKL with the receptor activator of NF-kB (RANK), present on the surface of osteoclastic precursor cells, stimulates the osteoclastic differentiation from monocyte or macrophage progenitors. Osteoprotegerin (OPG) is a member of the tumor necrosis factor (TNF) receptor superfamily, which prevents RANK mediated activation of osteoclastic differentiation by binding to RANKL^29^.

Periodontal ligament fibroblasts are known to contribute to osteoclast formation and promote destructive inflammatory periodontal diseases^28^. Interactions with bacteria or mechanical loading play a key role during this process. Moreover, Uchiyama, et al.^31^ (2009) has shown that dental pulp and periodontal ligament cells support the differentiation and function of osteoclasts. Recently, Inamitsu, et al.^16^ (2017) reported that 2-hydroxyethyl methacrylate (HEMA) and TEGDMA inhibited the osteoclast differentiation through different signaling pathways in bone marrow-derived macrophages (BMMs) and murine monocytic cell line RAW-D. The role of periodontal ligament and pulp cells on osteoclastogenesis and the effects of different resin monomers on osteoclast formation have been shown in various studies. However, there are no studies so far on the odontoclastogenic activity of pulp cells in the presence of TEGDMA. The null hypothesis is that TEGDMA has no significant effect on the odontoclastic differentiation ability of human dental pulp cells. Therefore, this study was carried out to determine if TEGDMA promotes the odontoclastic differentiation ability of human dental pulp cells (hDPCs). Thus, we evaluated the effects of TEGDMA on the ability of hDPCs to produce odontoclasts in the presence of CD14^+^ odontoclastic precursor cells derived from human peripheral blood.

## Material and Methods

This study was conducted in full accordance with the World Medical Association Declaration of Helsinki and was approved by the Local Ethics Committee of the Gulhane Military Medical Academy (32/2014). All participants provided informed consent forms.

### Cell culture

Dental pulp tissues were obtained from the molars of five healthy patients undergoing orthodontic treatment. The extracted molars were kept in phosphate-buffered saline solution (PBS, Biological Industries, Kibbutz Beit Haemek, Israel) containing 100 U/mL penicillin, and 100 μg/mL streptomycin (Biological Industries, Kibbutz Beit Haemek, Israel). After they were transferred to the laboratory, the extracted molars were cut horizontally at 1 mm below the cementoenamel junction. The pulp tissues were gently separated from the crown and root and placed in a 100-mm Petri dish. The pulp tissues were cut into small pieces with a sterile blade and cultured in ɑ-MEM (Gibco, Life Technologies, Grand Island, NY, USA) containing 10% fetal bovine serum (FBS; Biological Industries, Kibbutz Beit Haemek, Israel), 100 U/mL penicillin, and 100 μg/mL streptomycin (Biological Industries). Tissue cultures were maintained in a humidified atmosphere of 5% CO_2_ at 37°C. Cells from the fifth passage were used for the experiments.

### Generation of CD14^+^ cells

Histopaque-1077 (Sigma-Aldrich, St. Louis, MO, USA) was used to separate peripheral blood mononuclear cells (PBMC) from human peripheral blood by density-gradient centrifugation at 1800 rpm for 20 min. CD14^+^ cells were obtained from the PBMC with CD14 Microbeads (Miltenyi Biotec GmbH, Bergisch Gladbach, Germany) and SuperMACS^TM^ II Separator (Miltenyi Biotec GmbH, Bergisch Gladbach, Germany), in accordance with the manufacturer instructions.

### Generation of odontoclasts and designation of study groups

The following study groups were designed to analyze odontoclastic differentiation:

Human dental pulp cells (hDPCs);

hDPCs + CD14^+^ cells;

hDPCs + 1α,25(OH)_2_D_3_;

hDPCs + CD14^+^ cells + 1ɑ,25(OH)_2_D_3_;

hDPCs + CD14^+^ cells + TEGDMA;

hDPCs + 1ɑ,25(OH)_2_D_3_ + TEGDMA; and

hDPCs + CD14^+^ cells + 1ɑ,25(OH)_2_D_3_, +TEGDMA.

Each experimental group consisted of nine samples. hDPCs were seeded into 6-well plates at a concentration of 3×10^4^ cells/well, and cultured in ɑ-MEM (Gibco, Life Technologies, Grand Island, NY, USA) containing 10% FBS (Biological Industries, Kibbutz Beit Haemek, Israel), 100 U/mL penicillin, and 100 μg/mL streptomycin (Biological Industries, Kibbutz Beit Haemek, Israel). Upon achieving more than 80% confluency, CD14^+^ cells were used as odontoclast precursors and co-cultured with hDPCs at 2 x 10^5^ cells/well. The co-culture was performed in the presence or absence of 1α,25(OH)_2_D_3_ (10^−8^ M) (Sigma-Aldrich, St. Louis, MO, USA) and TEGDMA (0.1, 0.3, 1, and 3 mM). The concentration of the 1a,25(OH)_2_D_3_ was chosen from a previously reported study^21^. Culture conditions were maintained for 14 days in a humidified atmosphere of 5% CO_2_ at 37°C, with medium change every 3 days.

### Flow cytometry

Flow cytometry was used to evaluate odontoclastic differentiation after 14 days of incubation. After day 14, cells were suspended in 100 μL PBS, and stained with 10 μL anti-human CD51/CD61 mouse monoclonal antibody (BD Biosciences, USA), which bind selectively to odontoclasts, and incubated for 30 min at room temperature. After incubation, samples were centrifuged at 300 g for 5 min. Pellets were re-suspended with 400 μL PBS, and analyzed by flow cytometry (FACSDiva software, FACSAria, USA).

### TRAP staining

Cultures were fixed in 10% formalin neutral buffer solution (Sigma-Aldrich, St. Louis, MO, USA). After serial washing with PBS three times, odontoclasts were detected by staining for tartrate-resistant acid phosphatase (TRAP), a marker enzyme for odontoclasts, using a kit following the manufacturer's recommendations (Cosmo Bio Co., Ltd., Tokyo, Japan). TRAP activity was observed under an optical microscope with 63x magnification (Axio Vert. A1, Zeiss, Göttingen, Germany) and TRAP-positive cells were counted.

### Apoptosis assay

hDPCs were seeded into 6-well plates at 5 x 10^5^ cells/well density and exposed to serial dilutions of TEGDMA (0.1, 0.3, 1, and 3 mM) for 24 h. Apoptosis in hDPCs was analyzed by flow cytometry using an Annexin-V-FLUOS Staining Kit (Roche, Mannheim, Germany) following the manufacturer's instructions. Each experimental group consisted of nine samples.

### Quantitative Real-time PCR (qRT-PCR) analysis

hDPCs were seeded into 6-well plates at 1×10^6^ cells/well and incubated for 24 h in a humidified atmosphere of 5% CO_2_ at 37°C (n = 3 *per* experimental group). The cell cultures were exposed to serial dilutions of TEGDMA (0.1, 0.3, and 1 mM) for seven days. Total RNA was extracted from cultured hDPCs using an RNA isolation kit (High Pure RNA Isolation Kit, Roche, Mannheim, Germany), and cDNA was synthesized from 100 ng of total RNA using the Transcriptor High Fidelity cDNA Synthesis Kit (Roche, Mannheim, Germany). The cDNA obtained was used as a template for PCR.

Human RANKL and OPG mRNA levels were analyzed to evaluate the effects of TEGDMA on odontoclastic differentiation capability of hDPCs. Glucose-6-phosphate dehydrogenase (G6PD) was used as a housekeeping gene to normalize and determine the relative expression levels of RANKL and OPG. Probe-primer pairs for target genes were purchased from Roche Diagnostic as RealTime ready assays, i.e. RANKL (Cat. no. 144633), OPG (Cat. no.142904), and G6PD (Cat. no.102098). Real-time PCR was performed using the FastStart Essential DNA Probes Master (Roche, Mannheim, Germany) and a LightCycler^®^ 480 Instrument II (Roche, Mannheim, Germany). Real-time PCR conditions were as follows: denaturation at 95°C for 10 min, followed by 45 cycles of 95°C for 10 s, 60°C for 30 s, 72°C for 1 s, and cooling at 40°C. The mRNA level in each sample was calculated using the “ΔΔCT” method. Each experiment was performed in triplicate.

### Enzyme-linked immunosorbent assay (ELISA)

hDPCs were seeded into 6-well plates at 1×10^6^ cells/well and incubated for 24 h in a humidified atmosphere of 5% CO_2_ at 37°C (n = 9 *per* experimental group). The cell cultures were exposed to serial dilutions of TEGDMA (0.1, 0.3, and 1 mM) for seven days. Protein concentrations of OPG and RANKL in the cell culture supernatant were analyzed by ELISA. The ELISA kits (MyBioSource Inc., San Diego, CA, USA) employed a sandwich ELISA technique containing a capture antibody and label antibody. All steps in the manuals were followed during the analysis and results were read on an ELISA plate reader (Reader ELx 800, Biokit, Barcelona, Spain). The intra-assay precision was ≤8% and inter-assay precision was ≤12% for RANKL (while they were stated as <15% for OPG in the kit manual).

### Statistical analysis

The Kolmogorov-Smirnov test of distribution indicated that data for anti-CD51/CD61 positivity, TRAP positivity, apoptosis ratio, mRNA, and protein data for RANKL and OPG were skewed. Thus, results were summarized as medians and interquartile ranges. The Kruskal-Wallis test was used to compare three or more groups. The level of significance (p-value) was established at 0.05. SPSS Statistical Package Version 20.0 (IBM, Chicago, IL, USA) was used for all calculations.

## Results

### Analysis of odontoclastic differentiation

**Figure 1:**
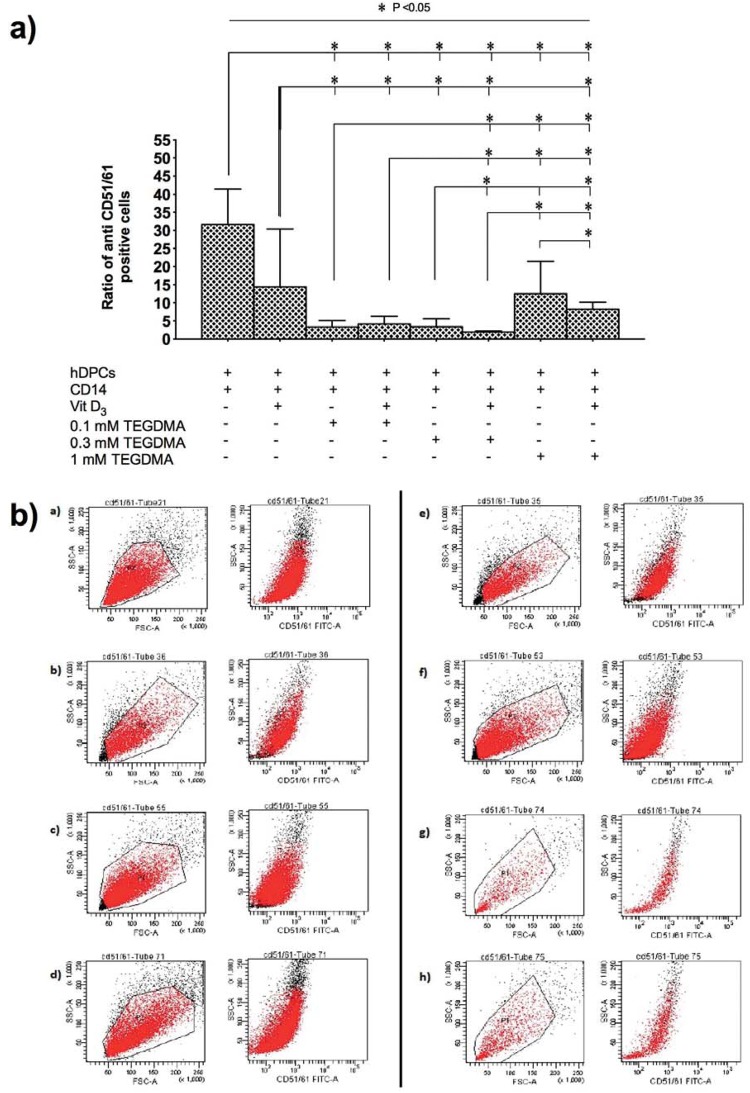
Ratio of antiCD51/61 positive cells generated by the co-culturing of different parameters was examined by flow cytometry analysis at the end of 14 days of incubation period. a) Graphs show the mean±SD per group. Significant differences between study groups are indicated by asterisk (p<0.05); b) One representative picture of flow cytometry analysis [a) hDPCs + CD14 b) hDPCs + CD14 + 1α,25(OH)_2_D_3_ c) hDPCs + CD14 +0.1 mM TEGDMA d) hDPCs + CD14 +0.1 mM TEGDMA + 1α,25(OH)_2_D_3_ e) hDPCs + CD14 + 0.3 mM TEGDMA f) hDPCs + CD14 + 0.3 mM TEGDMA + 1α,25(OH)2D_3_ g) hDPCs + CD14 + 1 mM TEGDMA h) hDPCs + CD14 + 1 mM TEGDMA + 1ɑ,25(OH)_2_D_3_]

Odontoclastic differentiation ratios in the different groups were analyzed after 14 days of incubation using flow cytometry (Figure 1a, 1b). Groups without CD14^+^ odontoclast precursor cells did not generate odontoclasts, as expected (data not shown). Co-culture of hDPCs and CD14^+^ cells (group ii) revealed the highest odontoclastic differentiation ratio. Addition of 1ɑ,25(OH)_2_D_3_ decreased the odontoclastic differentiation ratio, particularly in hDPCs + CD14^+^ cells + 0.3 mM TEGDMA and hDPCs + CD14^+^ cells + 1 mM TEGDMA groups (p<0.05). No such changes were seen in hDPCs + CD14^+^ and hDPCs + CD14^+^ cells + 0.1 mM TEGDMA groups (p>0.05). In general, the addition of TEGDMA lowered odontoclastic differentiation relative to groups without TEGDMA (p<0.05). Notably, 1 mM TEGDMA caused higher odontoclastic differentiation relative to 0.1 and 0.3 mM TEGDMA (p<0.05). No cell viability/odontoclastic differentiation was studied in the 3 mM TEGDMA group due to cytotoxicity (data not shown).

**Figure 2:**
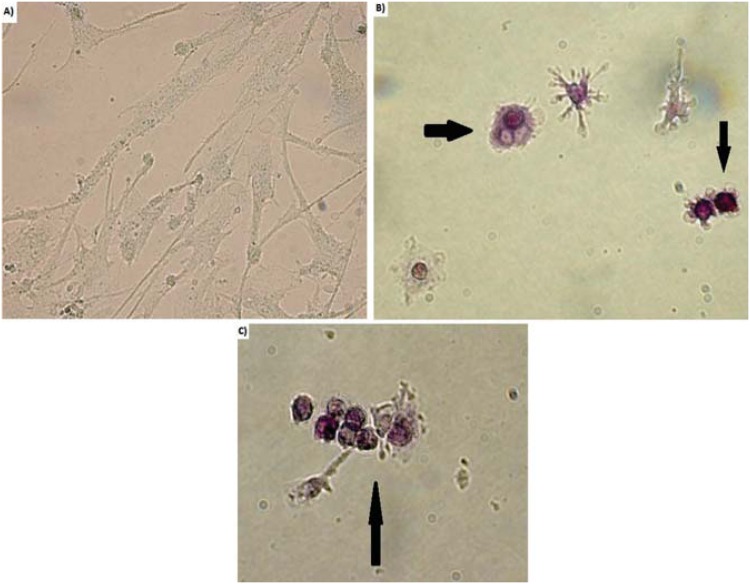
Analysis of odontoclastic differentiation by TRAP staining under an optical microscope with 63× magnification. TRAP-positive cells were observed through the co-culture of CD14^+^ cells and hDPCs at the end of 14 days incubation period. a) TRAP-negative b, c) TRAP-positive cells

Odontoclast cell formation was confirmed by counting TRAP-positive cells, which phenotypically assessed odontoclastic differentiation (Figure 2). The findings from the TRAP-positive cells generally correlated well with flow cytometry analysis (Table 1). However, 95±2% of TRAP-positive cells were mononuclear, and we did not detect any TRAP-positive cells in CD14^+^ negative groups.

**Table 1:** Number of TRAP-positive cells generated by the co-colturing of human CD14+ cells and hDPCs in the presence or absence of Vit D_3_ and TEGDMA

	Study Groups (n=9)	Median (Min-max)	*(p<0.05)
1	hDPCs + CD14	2465 (1065-3100)	1-2, 1-3, 1-4, 1-5, 1-6, 1-7, 1-8
2	hDPCs + CD14 + Vit D_3_	1043 (958-3742)	2-3, 2-4, 2-5, 2-6, 2-8
3	hDPCs + CD14 + 0.1 mM TEGDMA	260 (101-369)	3-6, 3-7, 3-8
4	hDPCs + CD14 + 0.1 mM TEGDMA + Vit D_3_	297 (98-456)	4-6, 4-7, 4-8
5	hDPCs + CD14 + 0.3 mM TEGDMA	276 (101-395)	5-6, 5-7, 5-8
6	hDPCs + CD14 + 0.3 mM TEGDMA + Vit D_3_	144 (108-165)	6-7, 6-8
7	hDPCs + CD14 + 1 mM TEGDMA	905 (760-1010)	7-8
8	hDPCs + CD14 + 1 mM TEGDMA + Vit D_3_	640 (586-750)	

* Study groups that reveal statistical differences from each other according to Kruskal-Wallis test

### Effect of TEGDMA on the apoptosis of dental pulp cells

**Figure 3:**
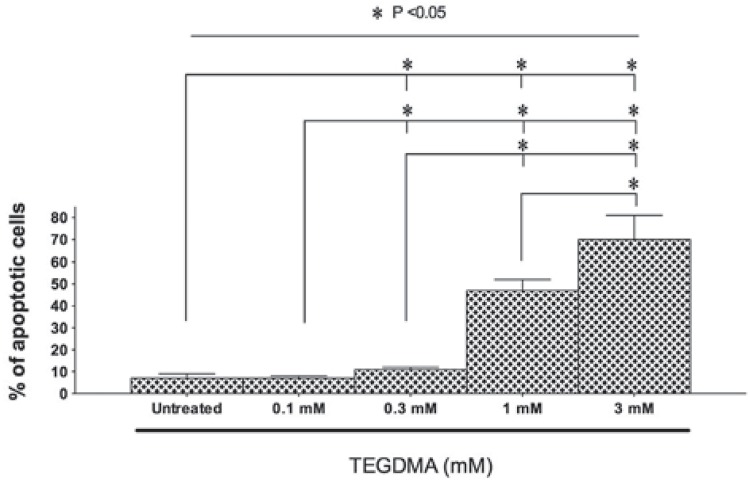
Apoptosis in hDPCs was induced proportionally with the concentration of TEGDMA. Data are expressed as means±SD. Significant differences between test materials and control group are indicated by asterisk (p<0.05)

hDPCs were exposed to 0.1, 0.3, 1, and 3 mM TEGDMA for 24 h, and it was found that TEGDMA dose-dependently induced apoptosis (Figure 3). Except for the lowest dose of 0.1 mM TEGDMA, the higher doses of 0.3, 1, and 3 mM TEGDMA increased apoptosis in comparison to the control group (p<0.001). Furthermore, there was a dose-dependent effect of TEDGMA on apoptosis induction in hDPCs.

### Expression of RANKL and OPG mRNA in dental pulp cells

**Figure 4:**
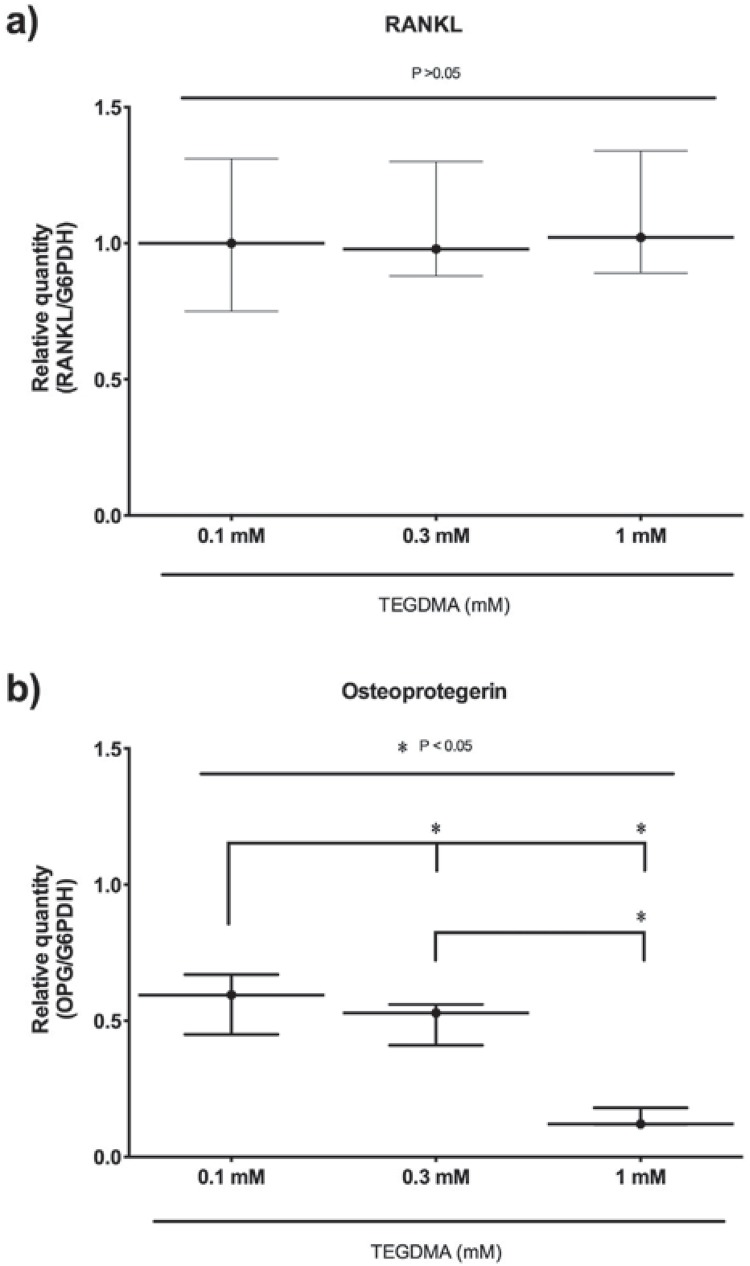
Effects of TEGDMA on RANKL (a) and OPG (b) mRNA levels in TEGDMA treated hDPCs at the end of 7 days of incubation period. Significant differences between study groups are indicated by asterisk (p<0.05)

No differences were observed among the TEGDMA containing groups (0.1, 0.3, 1) in terms of RANKL mRNA levels after 7 days of incubation (p>0.05) (Figure 4a). However, OPG mRNA levels were lower in the TEGDMA containing groups relative to the control group, and TEGDMA treatments demonstrated a dose-dependent decrease (p<0.05) (Figure 4b). RANKL and OPG mRNA levels could not be detected in the 3 mM TEGDMA group due to cytotoxicity (data not shown).

### RANKL and OPG protein levels in dental pulp cells

**Figure 5:**
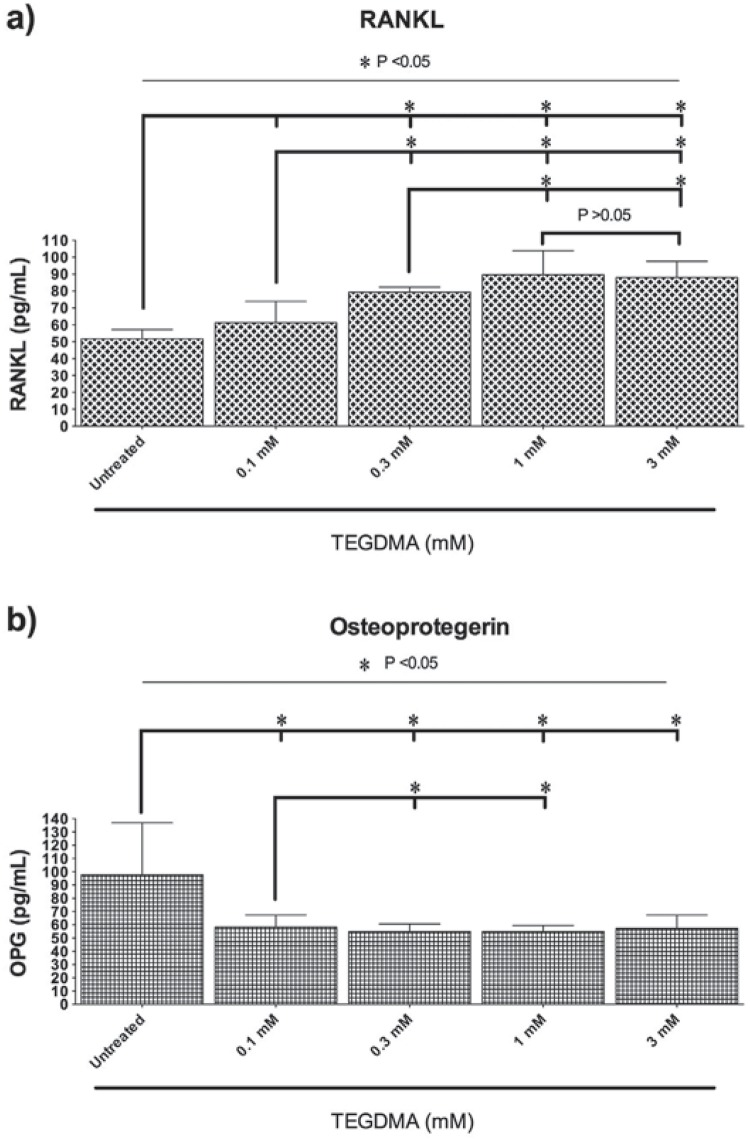
Effects of TEGDMA on RANKL (a) and OPG (b) protein levels in TEGDMA treated hDPCs at the end of 7 days of incubation period. Significant differences between study groups are indicated by asterisk (p<0.05)

RANKL protein levels were higher in the TEGDMA treated hDPCs regarding the control group (p<0.05) (Figure 5a), and TEGDMA treatments demonstrated a dose-dependent increase. There was a statistically significant difference between the groups, except for 1 and 3 mM TEGDMA containing groups (p<0.05).

OPG protein levels were lower in all TEGDMA treated groups regarding the control group (p<0.05) (Figure 5b). In particular, the 0.1 mM TEGDMA group had relatively lower OPG protein levels compared to 0.3 and 1 mM TEGDMA groups (p<0.05). There were no significant differences between the other test groups (p>0.05).

## Discussion

Resin monomers cause adverse biological effects by modulating different regulatory cellular mechanisms. Previous studies have shown that TEGDMA can disrupt mineralization capacity leading to cytotoxicity in hDPCs^1^ ^,^ ^6^ ^,^ ^11^ ^,^ ^18^ ^,^ ^20^. Recently, it has been shown that dental pulp cells support the differentiation and function of odontoclasts^31^. However, the effect of TEGDMA on the ability of dental pulp cells to induce odontoclastic differentiation has not yet been determined. In this study, our aim was to evaluate the cellular processes related to odontoclastic differentiation in TEGDMA-treated hDPCs. We anticipated that any information about the effects of TEGDMA on odontoclastic differentiation would provide a better understanding on the adverse effects of TEGDMA.

In this study, we attempted to determine the effects of TEGDMA on the generation of odontoclasts. This was carried out by co-culturing hDPCs and CD14^+^ monocytes derived from human peripheral blood. We conducted experiments in the presence or absence of 1α,25(OH)_2_D_3_, followed by incubating with serial dilutions of TEGDMA. Our results indicate that flow cytometry analysis using anti-human CD51/CD61 mouse monoclonal antibody was consistent with TRAP staining. However, TRAP-positive multinuclear cells constituted only 5±2% of all TRAP-positive cells. Previously, Domon, et al.^10^ (1997) reported that both mononuclear and multinucleated odontoclasts participate in tooth eruption, and further suggested that not all odontoclasts are multinucleated cells. Further, Hattersley & Chambers^13^ (1989) suggested that osteoclasts are initially mononuclear and might remain so, but later they become multinucleated. Moreover, odontoclastic cells can become multinucleated by cell fusion, and the formation of multinucleated odontoclasts has been described as an instantaneous event^10^. However, this study failed to observe a high proportion of multinuclear TRAP-positive cells and most of the odontoclasts were mononuclear. Although we do not have any evidence, we speculate that short culture time and higher biological activity of TEGDMA may have contributed to the high proportion of mononuclear TRAP-positive cells. Further, extending the culture period may increase the fusion of mononuclear cells.

The study groups without CD14^+^ cells did not reveal any odontoclastic differentiation. This observation was consistent with a previously reported study and reinforces the fact that odontoclastic precursor cells are required for odontoclastic differentiation^31^. Co-culture of hDPCs and CD14^+^ cells led to the highest odontoclastic differentiation ratio, and an addition of TEGDMA to the culture medium reduced odontoclastic differentiation ratios. Notably, the observed suppressive effect of TEGDMA was attenuated at higher concentrations. A reduction in the odontoclastic differentiation in the presence of lower doses of TEGDMA indicates that treatment with dental materials containing resin monomers may diminish the potential to induce odontoclastic resorption. However, a dose-dependent increase in the odontoclastic differentiation suggests that circumstances that lead to an increase in the release of resin monomers into oral environment, such as mechanical/chemical degradation or inadequate polymerization, may increase the possibility of odontoclastic resorption. Based on our observations, further studies are essential to explore the clinical effects of TEGDMA related odontoclastic differentiation.

It is known that 1α,25(OH)_2_D_3_ plays a key role in both odontoblastic and odontoclastic differentiation^4^. Normal levels of vitamin D and its active metabolite 1α,25(OH)_2_D_3_ are important for bone mineralization. However, increased 1α,25(OH)_2_D_3_ enhances bone degradation by acting on osteoblasts, causing them to release RANKL, which in turn activates osteoclasts. Besides that, Kim, et al.^17^ (2013) reported that 1α,25(OH)_2_D_3_ inhibits the osteoclast differentiation by suppressing the expression of RANK in the human peripheral blood osteoclast precursors. Although methodologies and applied concentrations of 1α,25(OH)_2_D_3_ are different, this study is generally consistent with the aforementioned study^17^. This study revealed that generation of odontoclasts was significantly suppressed by 1α,25(OH)_2_D_3_ in most of the study groups at a concentration of 10^−8^ M. This result indicates that 10^−8^ M of 1α,25(OH)_2_D_3_ causes a decrease in the odontoclastic differentiation ability of hDPCs, and may further suppress potentially adverse biologic effects of TEGDMA. On the contrary, Takahashi, et al.^30^ (2014) reported that 1α,25(OH)_2_D_3_ induces bone resorption and osteoclastogenesis by stimulating RANKL expression. We think that differences related to experimental design, cell types used, applied concentrations of 1α,25(OH)_2_D_3_, and differential biological activity of TEGDMA on dental pulp tissue may help explain the contradictory results. Even though we could not provide any evidence of a direct relationship between different 1α,25(OH)_2_D_3_ concentrations and quantity of odontoclastic differentiation, we speculate that dose changes in 1α,25(OH)_2_D_3_ may promote the odontoclastic differentiation ability of hDPCs based on previous reports. Therefore, further studies are necessary with different doses of 1α,25(OH)_2_D_3_ to evaluate the phenotypic effects of TEGDMA on odontoclastic differentiation.

RANKL plays an important role in bone regeneration and remodeling by activating osteoclasts/odontoclasts. Previous studies have shown that TEGDMA is a biologically active chemical and interacts with living cells by influencing different biological pathways^8^. Although TEGDMA did not affect the expression of RANKL mRNA, the RANKL protein was increased by TEGDMA in hDPCs. Therefore, TEGDMA may be affecting post-transcriptional regulation of gene expression. Notably, RANKL seems to be an important regulatory factor in odontoclastic differentiation of TEGDMA treated hDPCs. OPG is known as the main inhibitor of the osteoclastic/odontoclastic differentiation process. Our results indicate that TEGDMA decreased the expression of OPG mRNA and protein in a dose-dependent manner. This effect was consistent with our findings from flow cytometry and TRAP analysis, which indicated an increase in odontoclastic differentiation with an increased dose of TEGDMA. However, when all results were taken into account, there seems to be a discrepancy between them. Increase of RANKL and decrease of OPG proteins are supposed to induce odontoclastic differentiation. However, the number of anti-human CD51/CD61 and TRAP positive cells were decreased by TEGDMA treatment, compared to control, as shown in Figure 1 and Table 1. In this study, we examined the effects of TEGDMA on the RANKL and OPG proteins, which were upstream of the odontoclastic differentiation pathway,. However, there are several downstream signaling pathways related to odontoclastic differentiation such as NFATc1, NFkB, Akt, ERK, JNK, and p38 MAPKs^5^ ^,^ ^9^ ^,^ ^23^ ^,^ ^33^. Additional effects of TEGDMA on these different signaling pathways may contribute to the effects of TEGDMA on the odontoclastic differentiation ability of hDPCs and may be the cause of this inconsistency between the results.

Recently, Inamitsu, et al.^16^ (2017) reported that HEMA and TEGDMA inhibited the osteoclast differentiation in BMMs and RAW-D cells. Furthermore, the study also showed that NFATc1, ERK, and JNK signaling pathways played a key role in osteoclastogenesis for the HEMA, whereas NFATc1, Akt, and JNK pathways associated with the TEGDMA mediated osteoclastogenesis. The results seem similar in some aspects and support our findings. However, there are some discrepancies between the two studies. Unlike this study, Inamitsu, et al.^16^ (2017) reported a decrease in the osteoclastic differentiation of BMMs with the increasing dose of TEGDMA in TRAP analysis. However, odontoclastic differentiation ratios revealed an augmentation with increasing dose of TEGDMA in this study. Differences between the experimental designs may be the cause of the seemingly conflicting results. Inamitsu, et al.^16^ (2017) counted only the cells with three or more nuclei as TRAP-positive, whereas we took into account all the mononuclear and multinucleated cells during the TRAP analysis. Cell types and duration of the culture time used to monitoring osteoclastic differentiation were also different and this phenomenon may be an important factor for the osteoclastic differentiation of the cells. Furthermore, Inamitsu, et al.^16^ (2017) showed that TEGDMA has slight cytotoxic effects on BMM-derived osteoclasts. When we examined the results of the study, cytotoxicity was found to be mild at doses up to 1 mM, but cytotoxicity gradually increased at doses between 1 and 2 mM. Moreover, Inamitsu, et al.^16^ (2017) did not test the more cytotoxic doses of TEGDMA such as 3 mM as this study. It is to be noted that in the presence of a high dose of TEGDMA (3 mM) no results were obtained by flow cytometry, TRAP staining, qRT-PCR, or ELISA analyses. Consistent with previous studies indicating that TEGDMA can activate apoptotic pathways^3^ ^,^ ^15^, this study revealed that TEGDMA caused a dose-dependent increase in apoptosis of hDPCs and cell viability has been adversely affected in the presence of high doses of TEGDMA. Therefore, it has not been possible to carry out studies on the effects of high doses of TEGDMA on odontoclastic differentiation ability of hDPCs.

Resin composites are widely used in dentistry and many factors such as secondary caries, defects in restorations, marginal degradation, and pulpal death may cause the replacement of restorations or fail of the treatment. A detailed understanding of biological effects of resin monomers is important to increase treatment efficiency and prevent the need of endodontic treatment or loss of the tooth. Therefore, the inhibitory effects of resin monomers on odontoclastic differentiation ability of hDPCs may help understand the effects of resin monomers on the longevity of restorations and treatment success.

## Conclusion

We found that TEGDMA reduces the odontoclastic differentiation ability of hDPCs. However, odontoclastic differentiation ratios augmented with increasing dose of TEGDMA.
